# Sensorized T-Shirt with Intarsia-Knitted Conductive Textile Integrated Interconnections: Performance Assessment of Cardiac Measurements during Daily Living Activities

**DOI:** 10.3390/s23229208

**Published:** 2023-11-16

**Authors:** Abdelakram Hafid, Emanuel Gunnarsson, Alberto Ramos, Kristian Rödby, Farhad Abtahi, Panagiotis D. Bamidis, Antonis Billis, Panagiotis Papachristou, Fernando Seoane

**Affiliations:** 1 Textile Materials Technology, Department of Textile Technology, Faculty of Textiles, Engineering and Business Swedish School of Textiles, University of Borås, 503 32 Borås, Sweden; emanuel.gunnarsson@hb.se (E.G.); alberto.ramos_serrano@hb.se (A.R.); kristian.rodby@hb.se (K.R.); fernando.seoane@ki.se (F.S.); 2 School of Innovation, Design and Engineering, Mälardalen University, 722 20 Västerås, Sweden; 3 UDIT—University of Design, Innovation and Technology, 28016 Madrid, Spain; 4 Institute for Clinical Science, Intervention and Technology, Karolinska Institutet, 141 83 Stockholm, Sweden; farhad.abtahi@ki.se; 5 Department of Medical Care Technology, Karolinska University Hospital, 141 57 Huddinge, Sweden; 6 Department of Clinical Physiology, Karolinska University Hospital, 141 57 Huddinge, Sweden; 7 Lab of Medical Physics and Digital Innovation, School of Medicine, Aristotle University of Thessaloniki, 541 24 Thessaloniki, Greece; bamidis@auth.gr (P.D.B.); ampillis@auth.gr (A.B.); 8 Academic Primary Health Care Center, Region Stockholm, 104 31 Stockholm, Sweden; panos.papachristou@ki.se; 9 Division of Family Medicine and Primary Care, Department of Neurobiology, Care Sciences and Society, Karolinska Institutet, 141 83 Stockholm, Sweden

**Keywords:** sensorized T-shirt, textile electrodes, activities of daily living, ECG, HR, wearable monitoring

## Abstract

The development of smart wearable solutions for monitoring daily life health status is increasingly popular, with chest straps and wristbands being predominant. This study introduces a novel sensorized T-shirt design with textile electrodes connected via a knitting technique to a Movesense device. We aimed to investigate the impact of stationary and movement actions on electrocardiography (ECG) and heart rate (HR) measurements using our sensorized T-shirt. Various activities of daily living (ADLs), including sitting, standing, walking, and mopping, were evaluated by comparing our T-shirt with a commercial chest strap. Our findings demonstrate measurement equivalence across ADLs, regardless of the sensing approach. By comparing ECG and HR measurements, we gained valuable insights into the influence of physical activity on sensorized T-shirt development for monitoring. Notably, the ECG signals exhibited remarkable similarity between our sensorized T-shirt and the chest strap, with closely aligned HR distributions during both stationary and movement actions. The average mean absolute percentage error was below 3%, affirming the agreement between the two solutions. These findings underscore the robustness and accuracy of our sensorized T-shirt in monitoring ECG and HR during diverse ADLs, emphasizing the significance of considering physical activity in cardiovascular monitoring research and the development of personal health applications.

## 1. Introduction

The field of wearable technology has experienced significant growth in recent years, with products that incorporate advanced sensors, algorithms, and wireless communication technologies to monitor a range of physiological parameters, including heart rate, physical activity, sleep patterns, and environmental factors [1]. These devices have various applications, including workplace safety, employee productivity monitoring, and health and wellness management, through continuous and non-invasive monitoring of vital signs and health metrics [2,3,4,5,6,7]. For example, some of the most popular wearable devices include smartwatches, fitness trackers, and medical-grade wearables that monitor the health status of patients [8,9,10].

Assessing cardiac health in clinical settings conventionally involves the measurement of important physiological parameters, such as electrocardiogram (ECG) and heart rate (HR). However, traditional electrodes used for ECG and HR monitoring typically require the use of conductive gels or adhesives to improve skin contact, as well as external cables to connect the electrodes to the device [11]; this setup can be uncomfortable for the wearer, and the adhesive or gel can cause skin irritation or rashes [11,12,13,14]. In contrast, wearable technology devices now offer the possibility of continuous and non-invasive monitoring of these parameters. However, there are still challenges associated with developing wearable sensors that are comfortable to wear while using materials that will not irritate the skin and that can provide accurate and reliable measurements [15,16,17].

Smart T-shirts incorporating textile electrodes have emerged as a promising solution for acquiring ECG and HR measurements in a comfortable and non-intrusive manner. These innovative T-shirts feature integrated textile electrodes seamlessly integrated into the fabric, thereby enabling accurate and reliable sensing of ECG signals and HR measurement and providing valuable insights into cardiovascular health. ECG biopotential waveforms acquisition is facilitated by the suitable contact of the textile electrodes with the wearer’s skin [11,18,19,20,21]. Moreover, the integration of miniaturized electronic modules within the smart T-shirt enables wireless transmission of the acquired ECG and HR data for analysis [22,23,24,25,26,27]. By leveraging advanced knitting or weaving techniques, the textile electrodes can be seamlessly integrated into the fabric, thereby preserving the T-shirt’s flexibility and stretchability for enhanced comfort during wear [25,28].

Recent advancements in textile technology have made possible to create innovative and fabrication-friendly methods of developing smart textiles. One of these methods involves integrating conductive yarns into the fabric to create intarsia-knitted patterns [29,30]. This integration facilitates a seamless interconnection between sensors and sensing electronics, reducing the need for additional conductive materials or external cables during physiological measurements. From a user perspective, the use of smart textiles, in the form of garments, has the potential to minimize any resistance or aversion to the use of non-essential wearables or accessories, despite the added functionality.

Understanding and investigating the influence of stationary and movement actions on ECG and HR measurements when using a smart T-shirt as a wearable sensor is vital for assessing reliability and improving personalized health monitoring solutions [31,32,33]. For that, activities of daily living (ADLs), such as sitting and standing, represent the stationary action category, while walking, mopping the floor, cleaning the house, and even running represent the movement action category [34]. ADLs are routine activities that individuals engage in, and they can have an impact on ECG and HR measurements [35]. The examination of ADLs will allow to understand how stationary and movement actions affect the accuracy and reliability of ECG and HR measurements obtained through the developed solution [36,37,38,39]. This investigation will contribute to a better understanding of the performance of the sensorized T-shirt in different activity scenarios and provide valuable insights for the development of wearable sensors for continuous cardiac monitoring during ADLs.

This study evaluates the performance of novel developed sensorized T-shirts with intarsia-knitted conductive interconnections, and it also investigates ADLs’ impact on ECG and HR measurements. A comprehensive set of experiments was simultaneously executed to record and compare ECG signals and HR using the sensorized T-shirts and a commercially available chest strap during various ADLs, including sitting, standing, walking, and mopping the floor. These are activities that represent common daily actions that individuals engage in, and they require different levels of physical exertion.

## 2. Materials and Methods

To obtain a comprehensive understanding of the impact of stationary and movement actions on ECG and HR measurements, we conducted an extensive investigation using a novel sensorized T-shirt. This study involved separate analyses for the stationary and movement categories, as well as for each specific daily activity.

### 2.1. Sensorized T-Shirt

The sensorized T-shirt was carefully crafted to ensure functionality, ergonomics, and data collection. In order to accomplish this objective, the sensorized T-shirt is composed of three different fabrics, each of which accomplishes a specific function.

The main fabric used is a single jersey structure made on a 28-gauge single bed Monarch single ring machine (Monarch Knitting Machinery Corp., Osaka, Japan). The single jersey was knitted with polyester 78/72 dtex plated with naked Lycra 78 dtex to enhance the fabric’s elasticity and increase the right fit to the body to obtain the best contact between body skin and textile’s main fabric.

The second fabric is a chest patch that contains an electrically conductive pathway and a pocket to store the ECG recording device. The Stoll ADF530-KI 7.2 flat knitting machine (Stoll, Reutlingen, Germany) was used for manufacturing this piece of fabric. With a configuration of a 14-gauge needle bed and 12-gauge needle hook, a pocket to fit the device was knitted using the half-gauge + intarsia technique for knitting two conductive paths to establish an electrical connection between the measuring leads and the device sensing port. The chest patch was knitted using the same polyester yarn we used for the main circular fabric cited before. However, instead of naked Lycra, a polyamide/Lycra 78/78 dtex yarn was used to improve durability. The material of choice for the conductive interconnecting paths and device connecting pads was a silver-plated polyamide conductive yarn, Statex 117 dtex.

The third and final fabric chosen for the development of the side electrodes of the sensorized T-shirt was Shieldex PBNII, a knitted fabric metalized with silver, which consists of 22% elastomer, making it stretchable on two sides and providing high conductivity and durability. It is also OEKO-Tex^®^-certified, and it can therefore be worn next to the skin.

As a result of combining the materials and knitting techniques discussed above, the garment seamlessly fit into the goal mentioned earlier in this section. The main circular knit fabric was composed of 90% polyester and 10% Lycra^®^, resulting in a final weight of 180 gsm and allowing for improved comfort and stretchability, which are essential for the right contact between the textile electrodes and the user’s skin. Because the conductive yarn and secondary knitted structure of the chest patch are stretchable and adaptable, the chest patch knitted with the Stoll ADF530-KI 7.2 Flat Knitting Machine ensures that the electrodes transmit data from the side electrodes to the device sensing port during ADLs. Finally, the Shieldex PBNII fabric used for the side electrodes provided superior conductivity, durability, and hypoallergenic skin properties, which are essential for a garment with such requirements.

**Figure 1:**
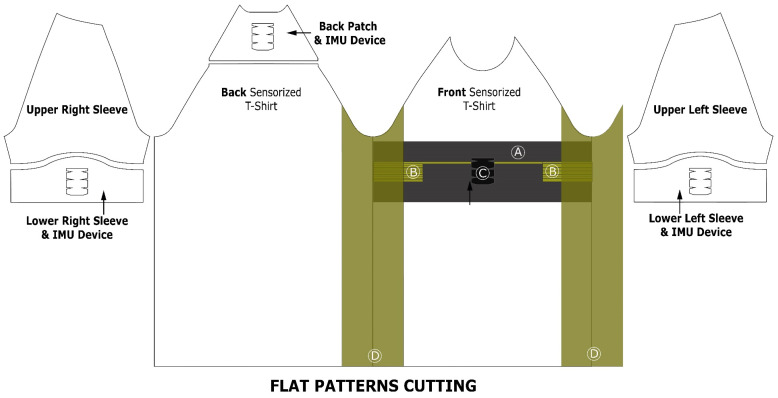
Flat patterns of fabrics to fabricate the sensorized T-shirt. Note that the side of the fabric facing the paper is the exterior of the T-shirt and the side facing the reader is the interior of the T-shirt.

Figure 1 shows all of the pieces required to manufacture the sensorized T-shirt prototype arranged as flat patterns. The intarsia-knitted piece, chest patch (A), and the two rectangular electrode pads (B) are sewn over the internal side of the main fabric of the front piece, resulting in a fully integrated front. From the electrode pads, two intarsia-knitted conductive tracks connect with two interconnecting pads (E) inside the ECG device pocket (C). The knitted pocket is meant to hold the sensing unit and establish the connection with the sensing ports of the ECG recorder. Overlapped to the chest patch and on both sides, it can see the textile electrodes (D) made of Shieldex PBN III fabric, which are sewn to the main fabric and chest patch. The portion of the side electrode fabric piece overlapping with the electrode pads on the chest patch is sewn with conductive yarn (Statex 117 dtex).

**Figure 2:**
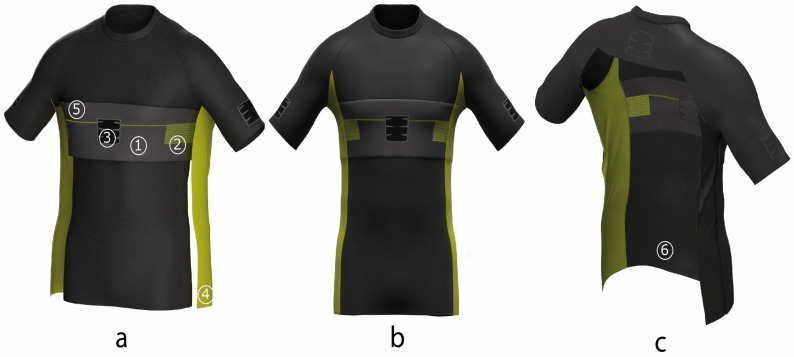
(**a**) Textile patterns from Figure 1 being assembled to fabricate the T-shirt (inside–out view). (**b**) T-shirt already fabricated (inside–out view). (**c**) Cross-sectional view of the T-shirt. The building elements of the T-shirt are the following: 1. intarsia-knitted chest patch, 2. chest rectangular electrode pad, 3. ECG device pocket, 4. side textile electrode, 5. interconnecting conductive track, 6. interior side of the T-shirt fabric.

Figure 2a illustrates the assembly sequence of the conductive pieces. Firstly, the chest patch (1) was sewn to the main fabric using two horizontal seams. The sewing machine used for this purpose was the Cover Stitch Machine (Juki, Ootawara, Japan) due to its bottom-chain stitch sewing system that preserves the stretchability required by the sensorized garment. The sewing machine used for this purpose was the four threads Overlock. This kind of machine is highly used in sport and fitness garments production. Finally, side electrodes (4) were sewn over the chest patch (1) and the main fabric using vertical seams made by the Cover Stitch Machine; two seams were used for every side, with one on the side front piece and another on the side back piece, thereby ensuring the side electrodes touch the chest sensors on both of the front sides. It is recommended that the side electrode piece is equally divided along both sides of the side seam at the front and the back.

Figure 2b shows the final result of the assembly process. Note that in both Figure 2a,b, the T-shirt is turned inside–out; therefore, the visible part of the T-shirt is the inside.

Figure 2c, through a cross-sectional view, shows the inside of the sensorized garment. This view allows one to see all of the conductive elements that take part in the manufacturing of the studied sensorized garment.

**Figure 3:**
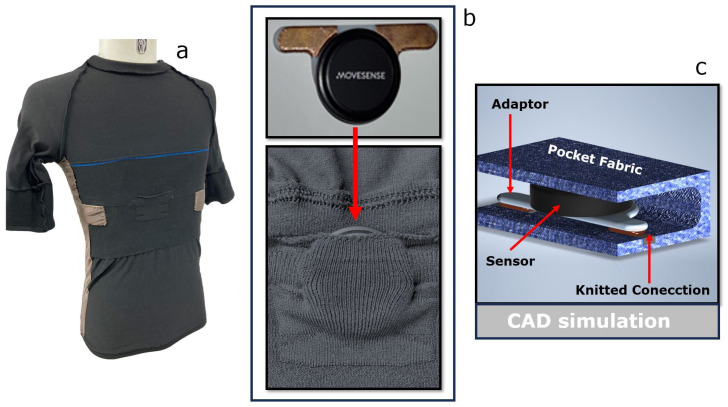
(**a**) Real sensorized T-shirt internal view turned around: central pocket, chest rectangular electrode pad, and side textile electrode. (**b**) The central pocket, zoomed in, housing the sensor and 3D-printed adaptor for friendly textile–electronic interconnection. (**c**) Detailed view of central pocket components.

Figure 3b,c illustrate the interior plugs of the chest pocket for the ECG recorder. This pocket has two narrow compartments right beneath the opening, with one such compartment going in each side direction. As a result, the pocket is, in principle, shaped like a “T”. Within those compartments, little plugs knitted with conductive yarns are used as connectors to the sensor + adaptor (schematically shown in Figure 3c). In Figure 3b, the copper-clad electrodes of the adaptor touch and secure contact with these knitted connection pads when the sensor is mounted in the pocket, thereby ensuring the connectivity and data collection of the ECG recorder.

The prolonged shape is used to exploit the stretching of the fabric and ensure the electrical interconnection between the sensor and the conductive textile pads. The nominal contact area with the adaptor, when mounted on the garment without the subject wearing it, is 0.5 cm^2^ (1 cm × 0.5 cm). When the garment is worn, this contact area may vary due to temporal changes in the fabric’s stretch. The compartments with the electrical contacts were designed to be smaller than the adaptor, thereby ensuring a consistent mechanical pressure between the contact members. While the exact pressure was not measured, impedance measurements between the sensor and the conductive yarn indicated an average of approximately 9.0 Ohms in the pre-stretch state and 9.9 Ohms when stretched with the T-shirt worn. The difference is insignificant and within range with the results presented in [40], where a detailed characterization of the resistance of the knitted textile conductive pathway was performed.

The knitting machines and fabrics used in the production process also ensured precision and accuracy in the arrangement of electrically conductive components. As a result of this careful selection and combination of fabrics and techniques, the T-shirt was not only functional, comfortable, and reliable in its measurements, but it could also be manufactured in an industrial garment production process.

### 2.2. Acquisition Device

**Figure 4:**
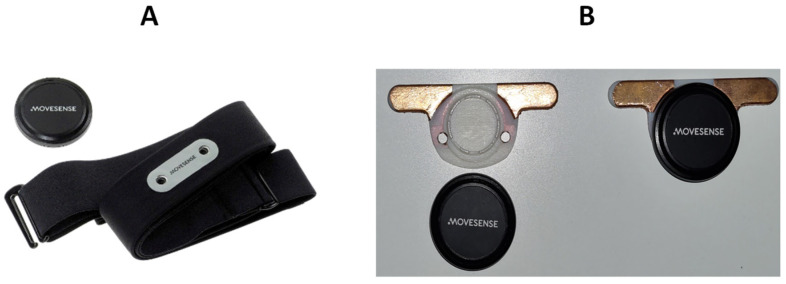
(**A**) Movesense Active with its chest strap delivered for ECG measurement. (**B**) Customized interface adaptor for Movesense Active device.

The Movesense Active device (Suunto, Vantaa, Finland) presented in Figure 4 was used for electrocardiographic measurements. The device can perform one-lead ECG measurements and directly calculate heart rate based on the ECG recordings. The Movesense Active device also includes an inertial measurement unit, magnetometers, and a gyroscope for additional sensing capabilities [41].

The Movesense Active device is a lightweight and compact device weighing only 10 g and measuring 36.60 mm in diameter and 10.60 mm in thickness. It is also water resistant up to 30 m with an IP68 rating. The manufacturer provides a chest strap with the device, which can be used for various wearable applications, such as walking, running, and swimming [41].

### 2.3. Experimental Measurement Setup

**Figure 5:**
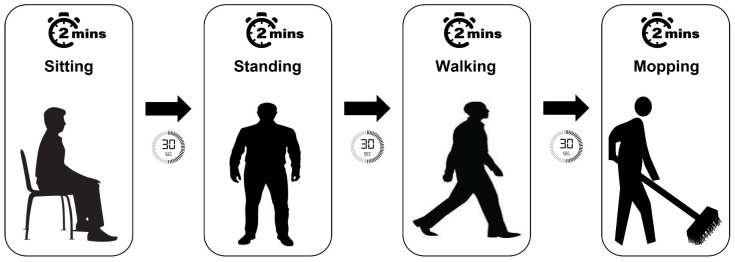
Diagram illustrating the experimental protocol and the ADLs selected.

In order to investigate the impact of various ADLs on ECG and HR measurements, a sequence of representative physical activities was chosen. This sequence included sitting, standing, walking, and mopping the floor, as such activities are common ADLs that individuals perform regularly. Each physical activity period lasted no less than 2 min, and one experimental round lasted for a minimum of 8 min. Figure 5 shows the experimental protocol followed.

This study was conducted at the laboratory for Textile-Electronics at the University of Borås, Sweden, and it involved six healthy male volunteers with a chest perimeter of 97 ± 9 cm and an age of 35 ± 9 years. Prior to the experiment, informed consent was obtained from all volunteers, including specific consent for the potential publication of identifying information/images in an online open access publication. All identifiable details, such as names and HIPAA identifiers, have been thoroughly removed from the manuscript. The methods employed in this study strictly adhered to the applicable guidelines and regulations. Ethical approval was granted by Sweden’s national ethical committee under the reference DNR 274-1, and all procedures were carried out in accordance with the principles outlined in the Declaration of Helsinki.

**Figure 6:**
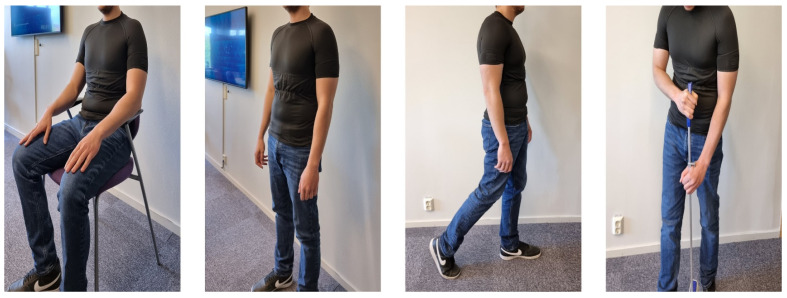
Volunteers wearing the tensorized T-shirt.

Figure 6 shows the complete system worn by a volunteer during each selected physical activity. Data were recorded via Bluetooth using the Movesense App (Suunto, Vantaa, Finland), which is accessible on IOS. The sampling frequency utilized was 128 Hz, thereby capturing both the ECG signal and HR measurements using the Movesense Active device.

### 2.4. Data Analysis and Pre-Processing

The data collected through the Movesense App were analysed and processed offline using a custom program implemented in MATLAB 2022 (The MathWorks, Inc., Natick, MA, USA). The ECG signals underwent no additional filtering during the processing or analysis. HR information was directly extracted from the Movesense App.

For the ECG recording, to assess the performance of the sensorized T-shirt, we employed various analytical methods. Shape Dynamic Time Warping (ShapeDTW), an extension of the Dynamic Time Warping technique [42], was chosen for the analysis. Each ECG signal acquired from the chest strap and sensorized T-shirt during the four different ADLs conducted by the volunteers were segmented by the PQRST complex. Subsequently, ShapeDTW was applied to each cycle pair from the chest strap and sensorized T-shirt. Then, the average ShapeDTW of the various cycles was calculated to evaluate the similarity between the ECG recordings obtained from the sensorized T-shirt and the four different ADLs performed by the volunteers.

Bland–Altman plots were used to assess any systematic bias or limits of agreement in HR measurements. This analysis provided insights into the consistency and comparability of the two methods. Additionally, violin plots were employed to visualize the distribution of HR differences, thereby enabling us to assess the variability and central tendencies of the data. Furthermore, the Mean Absolute Percentage Error (MAPE) was computed to quantify the average percentage difference between the two measurement configurations.

## 3. Results

More than 4000 s of recordings containing more than 6600 ECG complexes were obtained for this study.

### 3.1. ECG Signal Recording

**Figure 7:**
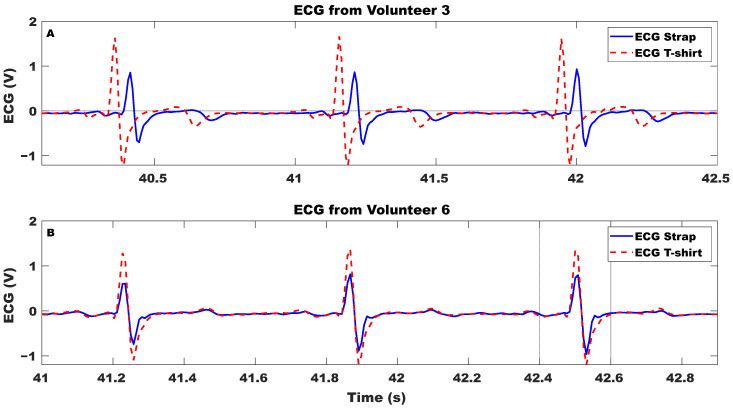
One-lead ECG simultaneously recorded using Movesense Active chest strap and sensorized T-shirt from two volunteers, (**A**) with 15 ms delay added for comparison purposes, and (**B**) both signals without additional delay.

**Figure 8:**
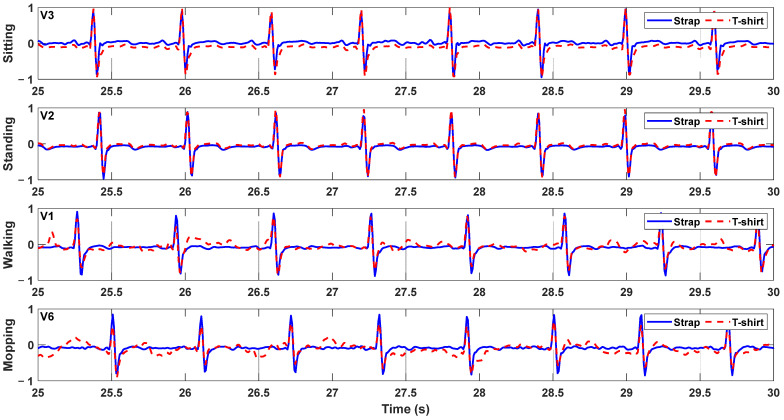
The 4 different plots represent the ECGs recorded in 4 different daily living activities in 4 different volunteers.

Figure 7 displays a three-cycle window demonstrating the ECG signals recorded simultaneously with a chest strap and a T-shirt. Figure 7A exhibits the recordings with a 15 ms delay to aid any visual comparison, while Figure 7B displays the recordings of another volunteer without delay to demonstrate complete synchronization. Figure 8 showcases four different plots, with each demonstrating an eight-cycle window of ECG readings during various daily living activities sourced from different volunteers. The recording acquired with the sensorized T-shirt is plotted with a discontinuous (- -) trace, while the recording acquired with the chest strap is plotted with a solid trace (-). The measurements indicate a high level of equivalence for all of the volunteers, with the primary distinction being the amplitude of the acquired signals. The average signal-to-noise ratios (SNRs) calculated for the chest strap and sensorized T-shirt are 40 and 25 dB, respectively, thereby exhibiting a good-quality ECG signal.

Table 1 presents the results of a comparison performed through the ShapeDTW for the recordings of all of the volunteers for each of the four different daily activities. The results obtained revealed distinct patterns across activities. Stationary tasks showed ECG ShapeDTW distances ranging from 1.61 to 6.42 mV, with a slight increase during standing that reports an average of 3.96 ± 1.28 mV for the 3.50 ± 2.05 mV reported during sitting. Walking displayed variations ranging from 4.70 to 10.48 mV, while the highest deviations occurred during mopping, with reported values between 5.44 mV and 14.05 mV.

The Mean ECG ShapeDTW distances for stationary tasks were 3.73 ± 0.23 mV, which are significantly lower than the value calculated from the recordings obtained during the activities with movement, which produce 8.77 ± 1.21 mV. Such an observed increase is expected because ECG recordings with textile electrodes are susceptible to movement artifacts.

### 3.2. Heart Rate

**Figure 9:**
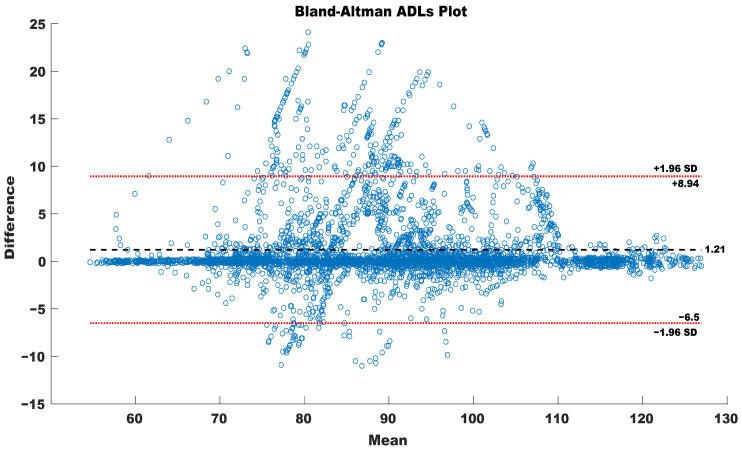
Bland–Altman of ADLs HR recorded with Movesense device for both chest strap and sensorized T-shirt.

Figure 9 illustrates the overall agreement with the Bland–Altman plot for the HR obtained from the Movesense Active device with the chest strap and sensorized T-shirt for all of the daily activities. The plot demonstrates a minor bias of 1.21 beats per minute (bpm) in the HR estimations, reflecting a robust agreement between the measurement techniques. Significantly, the narrow confidence intervals, spanning from −6.51 to +8.94 bpm, underscore the consistent bias, indicating a high level of agreement between the chest strap and the sensorized T-shirt.

**Figure 10:**
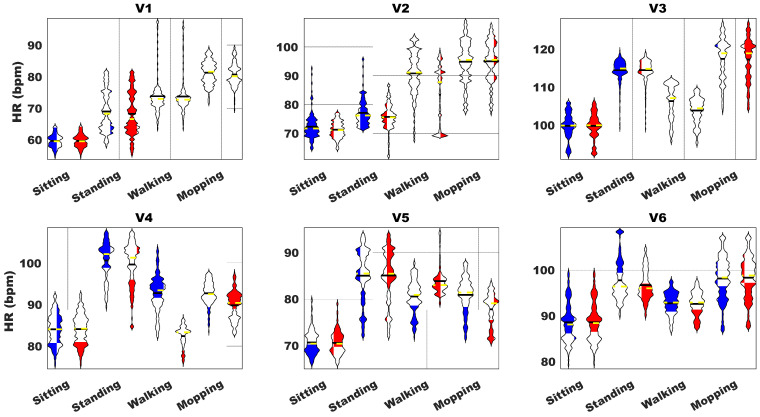
HR obtained from ADLs for both chest strap (blue), and sensorized T-shirt (red).

The violin plots presented in Figure 10 show the distribution of HR values categorized by activity for each of the volunteers. These plots serve as a comprehensive visual aid, providing a detailed overview of the complete data distribution and its associated probability density. The distribution indicates a notable consistency in the HR estimated values during stationary activities, reflecting the sensorized T-shirt’s ability to deliver reliable HR measurements during periods of minimal movement or rest. Moreover, this consistency extends to specific activities involving movement, such as walking and mopping, albeit with individual variations among the volunteers. These discrepancies may be attributed to distinctive physiological responses to physical activity or variations in the execution and intensity of the activities performed by the volunteers.

### 3.3. Activity of Daily Living

**Figure 11:**
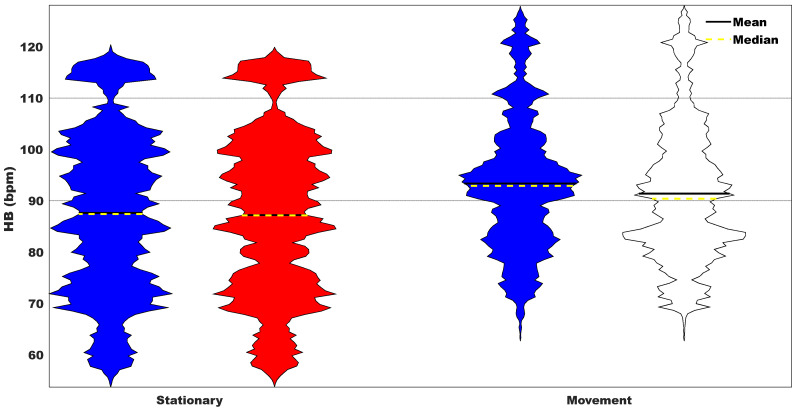
Distribution of HR obtained from all volunteers for the stationary and movement action. Blue represents the chest strap and red represents the sensorized T-shirt.

**Figure 12:**
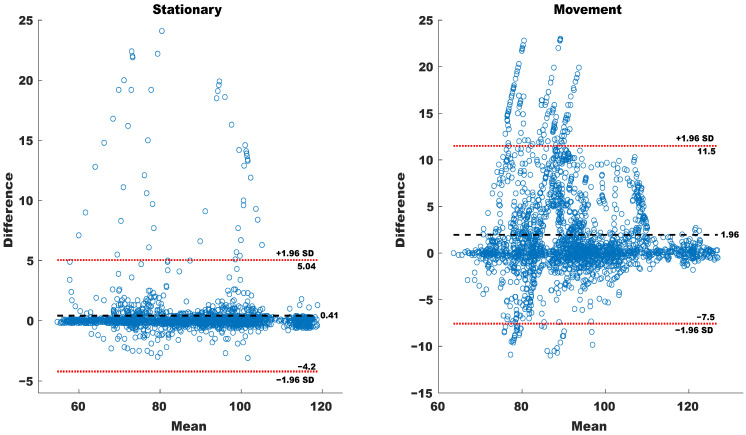
Bland–Altman of HR recorded for ADL stationary and movement actions.

The violin plots in Figure 11 and the Bland–Altman plot in Figure 12 group the HR estimation into two groups regarding the type of activity: stationary and movement. The larger level of agreement suggested by the distribution of HR showed by the violin plots is confirmed by the bias and the limits of confidence reported in the Bland–Altman plot. Furthermore, the actual MAPE between HR estimations obtained with the chest strap and the sensorized T-shirt for each of the volunteers for both categories (stationary and movement) is reported in Table 2, where it is possible to observe that in all but one volunteer, the value for the MAPE obtained for stationary activities is smaller than for movement activities.

Table 3 provides the MAPE between the HR obtained from the Movesense Active device using the chest strap and sensorized T-shirt for each volunteer and for each of the daily activities. The table shows that for all of the volunteers, the value of the MAPE is below 1% for sitting, while for standing it is four out of six, and it keeps decreasing with three out of six for mopping and only two out of six for walking. Notice that there is only one case where the value for the MAPE is over 5%.

## 4. Discussion

In recent years, sensorized T-shirts have gained increasing interest as wearable solutions for monitoring physiological signals during daily activities. Previous studies have explored the performance of sensorized T-shirts in capturing vital signs, such as ECG and HR, but often with a focus on a specific activity or with limited sample sizes [19,43,44], which may not fully represent common daily life scenarios [19,32,43]. In our study, we evaluated a novel sensorized T-shirt while encompassing a wide range of activities of daily living. By incorporating diverse ADLs, we obtained a more robust understanding of the measurement capabilities of the sensorized T-shirt, specifically regarding cardiac information and the impact of both stationary and non-stationary activities on measurement performance. Our findings contribute to the existing body of knowledge by demonstrating the effectiveness and versatility of the developed sensorized T-shirt across various ADLs. This study provides valuable insights into the measurement capabilities of the sensorized T-shirt, indicating its potential for global applicability and utilization in diverse situations and applications.

The prototype used for this study is not unisex, because it was principally developed for testing the functional feasibility of the intarsia knitting and large side electrodes. For such technical validation, it was assumed that gender did not play a role.

### 4.1. Measurement Settings and Sensorized T-Shirt

In the experimental settings of this study, the protocol intentionally disregarded the preparation of the skin–electrode interface, as is customary when performing this kind of study, and the ECG recorded signals were studied as is, i.e., without denoising or any other signal processing step for improving the acquired signal, including recordings that exhibited poor results, with the aim of accurately quantifying their impact on the measurements. The choice of using a large electrode design was motivated by the goal of improving the skin–electrode contact interface. Specifically, the large side electrodes have the same width regardless of the garment size: 5 cm. The lengths of the electrodes were 42, 44, 46, and 48 cm for sizes S, M, L, and XL, respectively, and they were strategically placed on the sides of the T-shirt, thus deviating from the initial attempt, which incorporated smaller textile electrodes measuring 4 × 5 cm. It is worth highlighting that the performance obtained with the current setup completely outclassed the prior design reported in [45], which could establish a good enough interface only to acquire an ECG long enough to establish a comparison during stationary conditions. This was the case for most of the activities, with the exception of sitting and in a limited number of volunteers. The fact that with the setup of side-tall electrodes it was possible to acquire ECG recordings of sufficient quality to enable a meaningful comparison underscores the importance of utilizing larger electrodes to maintain the required skin–electrode interface to be able to acquire proper ECG recordings across a wider range of activities and participants for both stationary and ambulatory conditions.

The manufacturing of the current prototypes required combining several fabrics with different functions. As a result of choosing circular knitted fabric for the T-shirt, the material functions as a second skin, allowing for very intensive use of the T-shirt. Despite circular knitting machines giving us the thinnest and lightest fabrics, they are unable to knit complex structures, such as those necessary for embedding electronics. This is where flat knitting machines come into play by enabling us to design a tailored pocket device with pathways integrated into a tubular structure all in one piece and with the same stretchability throughout the piece. We are aware that the choice of a finer gauge would have been better. However, the incorporation of Lycra^®^ filament through the plating knitting technique produced a fabric that matched perfectly with the main fabric regarding its behaviour, ensuring reliable signal collection. The matching provided by all three fabrics used in this project met our initial requirements and goals successfully, but a natural following step would be to test the feasibility of using a flat knitting machine with intarsia needles at the highest gauge possible to produce all of the textile elements in a single fabric: the pocket, the chest interconnecting piece, and textile electrodes. The difference in HR between volunteers during walking can be observed easily in Figure 10 in three out of the six volunteers, while in the case of mopping, it is only two of the volunteers presenting significant differences. These differences can be quantified in the value of the MAPE present in Table 2, where it is reported that the MAPE calculated is significantly larger in four of the volunteers during walking than during mopping.

### 4.2. ECG Measurement Performance

The ECG measurements obtained in our study revealed noticeable differences in amplitude between the chest strap and the sensorized T-shirt. These differences can be attributed to the distinct electrode placements employed by each wearable. Specifically, the strap used an electrode positioned on the front of the chest, approximately 13.5 cm away, while the sensorized T-shirt incorporated textile electrodes situated on the sides of the thorax. The distance between these electrodes varied between 40 and 60 cm, depending on the wearer’s size. It is important to consider that the amplitude of an ECG recording is influenced by the potential difference between the equipotential lines where the electrodes are placed, and this disparity tends to increase with greater electrode distances.

The calculated average ShapeDTW distances are equivalent to 0.37% and 0.87% of the observed amplitude variation for stationary and non-stationary activities, respectively, between the ECG signals obtained from the chest strap and the T-shirt. Such small values demonstrate a significant level of similarity between the recordings. The sensorized T-shirt consistently and accurately captures ECG signals during stationary actions, thereby ensuring the reliability of precise measurements. However, during movement actions, the results reveal an increasing trend in the average ShapeDTW distances corresponding to a high variability in the acquired ECG signal. This variability is likely the result of the movements affecting the garment’s fit to the body and the electrode placement and, consequently, the electrode–skin interface. While this difference is present between ECG acquisition during stationary activities and non-stationary activities, we would like to highlight that improvement in signal acquisition performance when comparing the T-shirt with a previous version without large side electrodes was significant [45].

In this study, our primary objective was to quantitatively investigate the performance of ECG measurements using our developed intarsia-knitted sensorized T-shirt with large side electrodes during various ADLs in normal, real-life conditions without implementing specific measures or actions to improve the interface between the electrode and the skin. This approach diverges from previous works, such as the referenced study [32], which focused on meticulous preparation and maintenance of a clean and stable skin interface to achieve optimal measurements. Moreover, it is important to address potential concerns regarding measurement quality impacted by the interface contact between the ECG device and the sensorized T-shirt within the knitted pocket. Investigations, including manual pressure and movement applied to the pocket area during ECG measurements, have consistently shown no discernible impact on signal quality, confirming the device–T-shirt interface’s meticulous design and optimization. Despite the encouraging technical results in several challenging aspects potentially influencing the quality of the signal acquisition, future developments should prioritize steps to further enhance the reliability and accuracy of ECG signal acquisition, including movement actions in ADLs. Maintaining a clean interface and minimizing motion artifacts, as reported in [32], are factors to consider in optimizing the sensorized T-shirt’s performance and usability for ECG monitoring during daily activities.

### 4.3. HR Measurement Performance

The analysis of HR measurements obtained from the Movesense Active device during various activities provides valuable insights into the performance of the sensorized T-shirt. The examination of HR distributions revealed consistent and equivalent values across a range of activities, thereby indicating reliable and accurate measurements. Notably, during stationary actions, the sensorized T-shirt demonstrated minimal differences in HR values, thus showcasing its effectiveness in capturing HR during periods of low intensity. Similarly, for movement actions, although slight variations in mean and median values were observed compared to the chest strap, the overall distribution patterns remained comparable.

To further validate these observations, Bland–Altman plots were utilized to assess the agreement between the sensorized T-shirt and the chest strap. The analysis revealed distinct levels of agreement for different activity categories. For stationary activities, the agreement between the two devices fell within an acceptable range, thus producing minimal measurement differences. However, during movement activities, a slightly wider range of measurement discrepancies was observed, indicating a potential impact on measurement accuracy. The larger discrepancies observed during walking and mopping were expected due to the movement of the arms during walking compared to the movement of the upper torso and arms simultaneously. The sets of pairs obtained for each of the volunteers suggest the periodic arm movements during walking might cause larger disturbances than the non-continued movements of the arms and torso during mopping.

Additionally, when considering all participants and activities collectively, the analysis demonstrated an acceptable level of agreement with some degree of variability. This highlights the importance of considering the various factors discussed earlier.

Furthermore, the high correlations observed between HR recordings from the sensorized T-shirt and the chest strap, along with the MAPE analysis indicating that 80% of the measurements had a maximum difference of ±3 beats per minute in HR, further solidify the functional equivalence between the two methods. These findings collectively emphasize the effectiveness of the sensorized T-shirt for accurate HR monitoring during diverse ADLs, thus establishing it as a promising wearable technology with broad applications. It is in the roadmap to investigate if the discrepancies observed in HR have an input in applications that might use Heart Rate Variability (HRV). For that, a larger cohort would be required, with long-lasting recordings.

### 4.4. Selection of Bottom-Chain Stitch Sewing Method for Garment Seams

**Figure 13:**
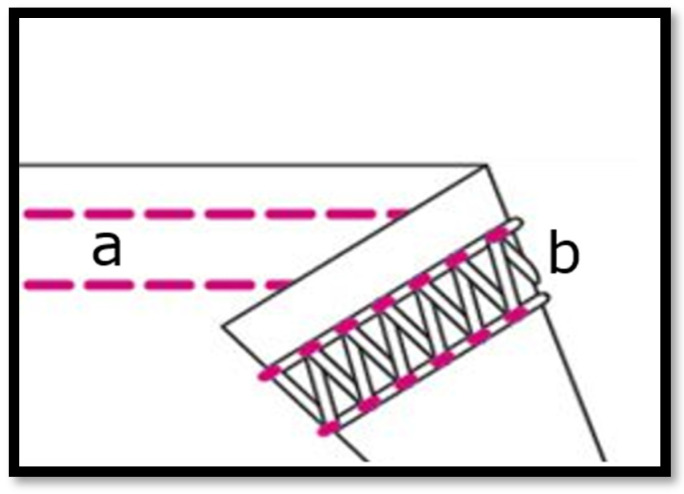
View of the cover stitch seam in detail. (**a**) Face side fabric by 2 needles work. (**b**) Under-side fabric view, displaying 2 needles work + hook work (multifilament textured threading).

The Coverstitch machine provides stretchy seams. For this garment, the two needles and three threads (stitch406) machine setup was used in accordance with ISO 4915 for elastic garments and double joining [46]. The third thread is a hook that makes the looper over the two needles. This seam structure allows the maximum stretchability of the main fabric due to the zigzag understructure, see Figure 13. In addition, the third thread used was a texturized multifilament yarn (instead of staple fiber yarn), which implies the best stretchability in the seam direction and gives high resistance and maximum elasticity to the multi-layer garment (main fabric + sensor patch + side electrodes). There is a significant influence of the choice of seam on the stretchability of the final garment to ensure the same stretch behaviour in all textile components and layers of the garment, thereby facilitating proper contact between the electrodes and the skin.

## 5. Conclusions and Future

In this study, we aimed to evaluate the feasibility of integrating intarsia-knitted conductive textile and strategically positioned larger electrodes within a sensorized T-shirt. The primary focus was to assess the functionality of this approach by targeting the specific technical requirements of the evaluation. Initially, we aimed to document the advantages of using larger electrodes from a measurement acquisition perspective. The integration of larger electrodes in the sensorized T-shirt was anticipated to enhance signal quality and minimize noise interference during HR monitoring, which was confirmed.

Additionally, to enrich the experimental study, a secondary objective was added: to evaluate the functional equivalence between the sensorized T-shirt and a commonly used chest strap in recording ECG signals and collecting HR data during four distinct ADLs. Simultaneous measurements demonstrated remarkable similarities in the ECG recordings, particularly in the QRS complexes, thereby confirming the functional parity between the sensorized T-shirt and the chest strap. Subsequently, we aimed to investigate the impact of ADLs on HR measurements. By analyzing the HR data obtained during various ADLs, we sought to comprehend the influence of different activities on HR measurements and evaluate the sensorized T-shirt’s performance under diverse conditions.

The evaluation of the obtained results indicates that the sensorized T-shirt developed in this study demonstrates comparable performance to the chest strap during both stationary and non-stationary activities. The average difference in HR for stationary actions was estimated to be less than 1%, while for movement actions it was below 3%, suggesting reasonably close performance between the sensorized T-shirt and the chest strap. These findings validate the successful achievement of the sensorized T-shirt’s intended objective, specifically in facilitating ECG and HR measurements during ADLs.

The novelty of this study lies in its comprehensive evaluation of a sensorized T-shirt with an intarsia-knitted conductive textile integrated for monitoring ECG and HR across a wide range of daily activities, thus providing valuable insights into its real-world applicability. Innovations include the strategic use of larger electrodes, optimizing the skin–electrode contact interface, and integrating multiple fabrics, including Lycra^®^ filament, to enhance signal reliability. Additionally, this study acknowledges the unique challenges posed by electrode placement and motion artifacts during movement actions, thereby setting a foundation for future improvements. Furthermore, the research highlights the sensorized T-shirt’s high consistency and accuracy in capturing HR data while emphasizing its potential as a reliable wearable technology for cardiovascular monitoring in various practical scenarios. Overall, this study’s holistic approach and innovative design considerations advance the field of smart textiles and wearable technology, thereby contributing to the broader adoption of such solutions in daily life health monitoring.

In future research, we will focus on comprehensively examining the impact of washing the T-shirt on its performance, evaluating the reliability of data, and conducting comparative analyses of data quality before and after washing.

Once the technical feasibility has been proven, the focus will be shifted to application, which will require the inclusion of female participants in the study. Such a study focusing on application will reach out beyond daily living activities to target working scenarios, and it will require a new T-shirt design customized to the workforce, for male and females, and fit for the requirements of the working environment.

Additionally, within the scope of the LifeChamps project, a real-life pilot study on a cohort of elderly cancer survivors was planned to be executed during spring 2023. The primary objective of this study is to assess the feasibility and efficacy of utilizing the sensorized T-shirt for long-term monitoring during the volunteers’ regular daily activities and engagement in various ADLs. By conducting this pilot study, an extensive dataset will be obtained, thereby significantly augmenting the possibilities for future solution development and refinement. The collected ECG recordings could feed the investigation regarding the input on HRV, because the pilot includes other sensors recording HR.

## Figures and Tables

**Table 1:** ShapeDTW for the ECG recordings for each volunteer for each ADL.

(mV)	Sitting	Standing	Walking	Mopping
V1	2.33	2.12	6.97	11.14
V2	1.70	4.05	10.48	7.02
V3	6.42	5.49	5.00	10.08
V4	1.61	3.00	9.20	14.05
V5	6.30	5.70	9.00	12.20
V6	2.68	3.44	4.70	5.44
Mean ± STD	3.50 ± 2.05	3.96 ± 1.28	7.55 ± 2.17	9.98 ± 2.95
	Stationary: 3.73 ± 0.23		Movement: 8.77 ± 1.21	

**Table 2:** MAPE of HR of each volunteer for the category of action.

(%)	Stationary	Movement
V1	0.73	0.53
V2	1.50	2.00
V3	0.26	1.37
V4	0.23	3.67
V5	0.19	4.49
V6	0.24	0.45
Mean ± STD	0.53 ± 0.47	2.17 ± 1.53

**Table 3:** MAPE of HR of each volunteer for each ADL.

(%)	Sitting	Standing	Walking	Mopping
V1	0.10	1.36	0.51	0.54
V2	0.29	2.71	3.68	1.31
V3	0.28	0.25	2.36	0.39
V4	0.17	0.29	4.24	3.09
V5	0.18	0.21	5.14	3.84
V6	0.12	0.37	0.53	0.37
Mean ± STD	0.19 ± 0.07	0.86 ± 0.92	2.74 ± 1.77	1.59 ± 1.38

## Data Availability

The datasets supporting the conclusions of this study are available upon request from the principal investigator (fernando.seoane@hb.se). The authors commit to providing access to the data and materials promptly to researchers with a qualified purpose in accordance with the ethical approval vetting the collection of the data.
